# Phase 1 pharmacokinetic and safety study of soticlestat in participants with mild or moderate hepatic impairment or normal hepatic function

**DOI:** 10.1002/prp2.1213

**Published:** 2024-07-11

**Authors:** Wei Yin, Pranab Mitra, Veronique Copalu, Thomas C. Marbury, Juan Carlos Rondon, Eric J. Lawitz, Valerie Lloyd, Mike Baratta, Mahnaz Asgharnejad, Tom Hui, Yasir Khan

**Affiliations:** ^1^ Takeda Development Center Americas, Inc. Cambridge Massachusetts USA; ^2^ Orlando Clinical Research Center Orlando Florida USA; ^3^ Clinical Pharmacology of Miami LLC Miami Florida USA; ^4^ The Texas Liver Institute University of Texas Health San Antonio San Antonio Texas USA

**Keywords:** hepatic function, hepatic impairment, pharmacokinetics, safety, soticlestat, TAK‐935

## Abstract

This phase 1, open‐label, three‐arm study (NCT05098054) compared the pharmacokinetics and safety of soticlestat (TAK‐935) in participants with hepatic impairment. Participants aged ≥18 to <75 years had moderate (Child‐Pugh B) or mild (Child‐Pugh A) hepatic impairment or normal hepatic function (matched to hepatic‐impaired participants by sex, age, and body mass index). Soticlestat was administered as a single oral 300 mg dose. Pharmacokinetic parameters of soticlestat and its metabolites TAK‐935‐G (M3) and M‐I were assessed and compared by group. The incidence of treatment‐emergent adverse events (TEAEs) and other safety parameters were also monitored. The pharmacokinetic analyses comprised 35 participants. Participants with moderate hepatic impairment had lower proportions of bound and higher proportions of unbound soticlestat than participants with mild hepatic impairment and normal hepatic function. Total plasma soticlestat pharmacokinetic parameters (maximum observed concentration [*C*_max_], area under the concentration‐time curve from time 0 to time of last quantifiable concentration [AUC_last_], and AUC from time 0 to infinity [AUC_∞_]) were approximately 115%, 216%, and 199% higher with moderate and approximately 45%, 35%, and 30% higher with mild hepatic impairment, respectively, than healthy matched participants. Moderate hepatic impairment decreased the liver's ability to metabolize soticlestat to M‐I; glucuronidation to M3 was also affected. Mild hepatic impairment resulted in a lower total plasma M‐I exposure, but glucuronidation was unaffected. TEAEs were similar across study arms, mild, and no new safety findings were observed. A soticlestat dose reduction is required for individuals with moderate but not mild hepatic impairment.

## Graphical abstract

As soticlestat is predominantly metabolized by the liver its clearance may be affected by hepatic impairment. This pharmacokinetic and safety study demonstrated that soticlestat dose reduction will be required in individuals with moderate but not mild hepatic impairment.

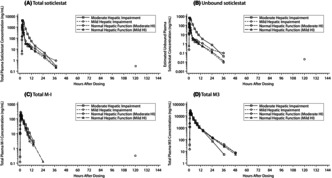

**Abbreviations**
AUC_∞_
: area under the total plasma concentration‐time curve from time 0 to infinity
AUC_last_
: AUC from time 0 to time of the last quantifiable total plasma concentration
BMI: body mass index
BP: blood pressure
CH24H: cholesterol 24‐hydroxylase
CI: confidence interval
CL/F: apparent clearance after extravascular administration, calculated using the observed value of the last quantifiable total plasma concentration
*C*_max_
: maximum concentration
CV%: arithmetic percent coefficient of variation
DEE: development and/or epileptic encephalopathy
DS: Dravet syndrome
ECG: electrocardiogram
GMR: geometric least squares mean ratio
LGS: Lennox–Gastaut syndrome
LSM: least squares mean
MPR: metabolite‐to‐parent ratio
QTcF: Fridericia's formula
SD: standard deviation
*t*_½z_
: terminal disposition phase half‐life
TEAE: treatment‐emergent adverse event
*t*_max_
: time of first occurrence of *C*_max_
UGT: uridine 5′‐diphospho‐glucuronosyltransferase
Vz/F: apparent volume of distribution during the terminal disposition phase after extravascular administration, calculated using the observed value of the last quantifiable total plasma concentration

## 1 INTRODUCTION

Epilepsy syndromes are characterized by specific clinical and electroencephalogram features. One subgroup of epilepsy syndromes are those associated with development and/or epileptic encephalopathy (DEE) or progressive neurologic deterioration.^1^ Dravet syndrome (DS) and Lennox–Gastaut syndrome (LGS) are both classified as DEE.^1^ DS is characterized by treatment‐resistant seizures and neurodevelopmental problems that start in infancy.^2^ LGS is associated with the onset of severe treatment‐resistant seizures in childhood as well as intellectual disability.^3^ Currently, DS and LGS are treated with generic antiseizure medications, with some adjunct therapies specifically licensed for DS and LGS, such as fenfluramine and cannabidiol, as well as several generic antiseizure medications.^4^, ^5^ However, seizure control remains a challenge.

Soticlestat (TAK‐935) is a novel inhibitor of cholesterol 24‐hydroxylase (CH24H)^6^ in phase 3 development as an add‐on therapy for the treatment of seizures associated with DS and LGS.^7^, ^8^ Cholesterol 24‐hydroxylase is a brain‐specific enzyme that converts cholesterol into 24*S‐*hydroxycholesterol, the latter being implicated as an endogenous modulator of *N*‐methyl‐D‐aspartate receptors that regulate excitatory synaptic function in the central nervous system as well as neuroinflammation.^6^, ^9^, ^10^ Preclinical studies in mouse models demonstrated the potential of soticlestat to reduce brain 24*S‐*hydroxycholesterol in a dose‐dependent manner, delay the onset of seizures, and reduce hyperexcitation, seizure burden, and number of seizures.^11^, ^12^, ^13^, ^14^, ^15^

Phase 1 studies in healthy volunteers established the pharmacokinetics and pharmacodynamics of soticlestat after single (up to 1350 mg) and multiple doses (100–600 mg/day).^16^, ^17^ The mean plasma maximum concentration of soticlestat (*C*_max_) was 43.5–7950 ng/mL, median time to *C*_max_ was 0.250–0.520 h, and terminal elimination half‐life was 0.820–7.16 h across doses ranging from 15 to 1350 mg.^16^ In these studies, all treatment‐emergent adverse events (TEAEs) associated with doses of up to 1350 mg as a single dose and 100–400 mg/day were mild or moderate, and dose dependently reduced plasma 24*S‐*hydroxycholesterol concentrations.^16^, ^17^

Model‐based simulations using available data determined 100–300 mg twice daily as the potential dose for phase 2 trials.^18^ In subsequent phase 1b/2a and phase 2 studies, participants with DEE, cyclin‐dependent kinase‐like 5 deficiency disorder, DS, or LGS had a reduction in seizure frequency. Furthermore, at doses of up to 300 mg twice daily, most TEAEs were mild or moderate in severity, and plasma 24*S‐*hydroxycholesterol levels were reduced.^19^, ^20^, ^21^

In vitro, plasma protein binding of soticlestat in humans was concentration‐dependent, ranging from 70.6% at 10 μg/mL, 93.4% at 1 μg/mL, and 94.0% at 0.1 μg/mL; soticlestat was mainly bound to human α‐1‐acid glycoprotein instead of human serum albumin. In healthy volunteers, soticlestat had an absolute bioavailability of 12.6%. There was near‐complete recovery of total radioactivity following a 300 mg dose of [^14^C]soticlestat: urine, 94.8%; feces, 2.7%. In urine, 85.9%, 0.1%, and 0.6% of the administered dose were recovered as TAK‐935‐G (M3), soticlestat, and metabolite M‐I, respectively.^22^ Soticlestat is predominantly metabolized by the liver via the glucuronidation pathway,^16^ with TAK‐935‐G (M3) being the major metabolite and M‐I being the N‐oxide metabolite.^18^, ^22^ Because hepatic impairment can affect the clearance of drugs metabolized by the liver,^23^ a pharmacokinetic study in individuals with hepatic impairment is recommended by the US Food and Drug Administration if hepatic metabolism and/or excretion account for a substantial portion (>20% of the absorbed drug) of elimination of the parent drug or active metabolite.^24^ This study aimed to assess the impact of hepatic impairment on the pharmacokinetics of a single dose of soticlestat and its metabolites M‐I and M3.

## 2 MATERIALS AND METHODS

### 2.1 Study oversight

All study documents were reviewed by the Advarra Institutional Review Board (Columbia, MD, USA) prior to study initiation. The study was approved by the local institutional review boards of the study sites and conducted in accordance with the Declaration of Helsinki, International Conference on Harmonization Harmonized Tripartite Guideline for Good Clinical Practice, and all applicable regulations. All participants provided written informed consent.

### 2.2 Study design and population

This was a phase 1, open‐label study of a single dose of oral soticlestat 300 mg with three treatment arms (NCT05098054)^25^: moderate hepatic impairment (Child‐Pugh B; score ≥7 and ≤9; anticipated enrollment *n* = 8); mild hepatic impairment (Child‐Pugh A; score ≥5 and ≤6; *n* = 8); and normal hepatic function (*n* = 12–24). Screening occurred at an outpatient visit up to 28 days before dosing. Participants were admitted to the clinic the day before dosing and remained until day 7. Follow‐up phone contact occurred 14 ± 2 days after dosing.

The inclusion criteria for all participants were age ≥ 18 to <75 years at screening; body mass index (BMI) ≥18.0 and ≤40.0 kg/m^2^ at screening and ≥50% of participants were required to have a BMI ≥18.0 and ≤35.0 kg/m^2^; sufficiently healthy for study participation based upon medical history, physical examination, vital signs, electrocardiogram (ECG), and screening clinical laboratory profiles, as deemed by the investigators or designees; supine pulse rate ≥40 and ≤99 beats per minute at screening; continuous nonsmoker or moderate smoker (≤10 cigarettes per day or the equivalent) before screening (participant agreed to consume no more than five cigarettes or equivalent per day from the 7 days prior to check‐in and until discharge); and agreement to comply with protocol contraceptive requirements.

For the hepatic impairment arms, additional inclusion criteria were supine blood pressure (BP) ≥80/40 (asymptomatic) and ≤150/95 mmHg at screening; QT interval corrected for heart rate using Fridericia's formula (QTcF) ≤500 ms and ECG findings considered normal or not clinically significant by the investigators or designees at screening; chronic hepatic impairment for ≥3 months before screening that was stable (no significant changes in hepatic function in the 30 days preceding screening or since the last visit if within 6 months before screening) and treated with stable doses of medication; and adequate renal function (creatinine clearance ≥50 mL/min) at screening. To assess hepatic impairment, two hepatic function assessments ≥48 h apart were required during the screening period unless a Child‐Pugh assessment score within 3 months prior to screening was available, in which case one assessment was conducted during screening. If the Child‐Pugh scores from both assessments indicated the same liver function category, soticlestat was administered as scheduled. If the results differed, a third assessment was conducted ≥24 h after the second. If the results of the second and third assessments agreed regarding the participant's liver function category, the participant was enrolled and received the day 1 dose within 48 h of the third assessment. If the second and third measurements differed, the participant was not eligible for the study.

Healthy participants were matched to hepatic‐impaired participants by sex (±2 per sex), age (mean ± 10 years), and BMI (mean ± 10%). In addition, healthy participants needed to have supine BP ≥90/40 and ≤150/95 mmHg at screening; QTcF ≤450 (males) or ≤470 ms (females) and ECG findings considered normal or not clinically significant by the investigators or designees at screening; liver function tests including alanine aminotransferase, aspartate aminotransferase, alkaline phosphatase, and total bilirubin at or below the upper limit of normal at screening and check‐in; and adequate renal function (creatinine clearance ≥60 mL/min) at screening.

The exclusion criteria for the study included history or presence of a clinically significant medical or psychiatric condition or disease (aside from hepatic impairment in the appropriate arms) or presence of psychotic disorders such as psychosis, delusions, or schizophrenia in the opinion of the investigators or designees; history of liver or other solid organ transplant (hepatic impairment arms); history or presence of alcoholism and drug abuse within the past 6 months (hepatic impairment) or 2 years (healthy participants) prior to dosing; positive result at screening for human immunodeficiency virus; positive result at screening for hepatitis B surface antigen or hepatitis C virus (HCV; healthy participants); positive for hepatitis B surface antigen with hepatitis B virus DNA ≥1000 copies/mL in the plasma (hepatic impairment); and positive for HCV antibodies with detectable HCV RNA in the plasma (hepatic impairment).

The moderate hepatic impairment arm was conducted first, followed by the matched normal hepatic function arm. After enrollment of the mild hepatic impairment arm, up to 12 additional participants could be enrolled for the matched normal hepatic function group to ensure a minimum of 12 participants with normal hepatic function and matching the mean of the mild hepatic impairment arm by age, sex, and BMI. Soticlestat was taken as three 100 mg immediate‐release tablets on day 1 with approximately 240 mL water under fasting conditions (≥10 h before dosing and 4 h post‐dose).

### 2.3 Study assessments and endpoints

Medical history and demographic data were recorded at screening. Twelve‐lead ECG, pulse rate, and BP were taken 2, 24, and 144 h after dosing or at early termination, and respiratory rate and temperature were taken 24 and 144 h after dosing or at early termination. Hematology, coagulation, serum chemistry, and urine analysis were conducted 48 and 144 h after dosing or at early termination. Blood samples for pharmacokinetic assessments of soticlestat and its metabolites M‐I and M3 were taken predose and 0.133, 0.25, 0.5, 0.75, 1, 1.5, 2, 4, 6, 8, 10, 14, 24, 36, 48, 72, 96, 120, and 144 h after dose administration or at early termination. Blood samples for plasma protein binding were taken predose and 0.5 and 10 h after dose administration. TEAE monitoring occurred throughout the study and at follow‐up.

The study's primary endpoints were the following pharmacokinetic parameters for soticlestat: *C*_max_; area under the total plasma concentration‐time curve from time 0 to infinity (AUC_∞_); and AUC from time 0 to time of the last quantifiable total plasma concentration (AUC_last_). The incidences of TEAEs and clinically significant abnormal values for laboratory evaluations, vital signs, ECG parameters, and Columbia‐Suicide Severity Rating Scale were secondary objectives. Exploratory endpoints were plasma pharmacokinetic parameters of soticlestat metabolites M‐I and M3 (*C*_max_, AUC_∞_, AUC_last_, metabolite‐to‐parent ratio [MPR] *C*_max_, MPR AUC_last_, and MPR AUC_∞_); additional plasma pharmacokinetic parameters of soticlestat and the metabolites M‐I and M3 (time of first occurrence of *C*_max_ [*t*_max_] and terminal disposition phase half‐life [*t*_½z_]); additional plasma pharmacokinetic parameters of soticlestat (apparent clearance after extravascular administration, calculated using the observed value of the last quantifiable total plasma concentration [CL/F]; apparent volume of distribution during the terminal disposition phase after extravascular administration, calculated using the observed value of the last quantifiable total plasma concentration [Vz/F]); and plasma protein binding of soticlestat: fraction of unbound soticlestat.

Total soticlestat, M‐I, and M3 concentrations and unbound soticlestat concentrations were determined using liquid chromatography–tandem mass spectrometry by CMIC, Inc. (Hoffman Estates, IL, USA). Unbound plasma soticlestat concentrations were measured using ultrafiltration (by CMIC, Inc.). Pharmacokinetic parameters were calculated using Phoenix^®^ WinNonlin^®^ version 8.3.4.

### 2.4 Statistical methods

Descriptive statistics were derived for the concentration data: number of observations (*n*), arithmetic mean (mean), standard deviation (SD), arithmetic percent coefficient of variation (CV%), standard error of the mean, minimum, median, and maximum. Pharmacokinetic parameter data for total plasma soticlestat, M‐I, and M3, and unbound plasma soticlestat were summarized by hepatic function arm using n, mean, SD, CV%, standard error of the mean, minimum, median, maximum, geometric mean (*C*_max_ and AUCs only), and geometric CV% (*C*_max_ and AUCs only). Continuous variables were summarized using *n*, mean, SD, minimum, median, and maximum. Analysis of variance was used to compare hepatic function groups, and all statistical analyses were conducted using SAS^®^ version 9.4.

### 2.5 Nomenclature of targets and ligands

Key protein targets and ligands in this article are hyperlinked to corresponding entries in http://www.guidetopharmacology.org, the common portal for data from the IUPHAR/BPS Guide to PHARMACOLOGY,^26^ and are permanently archived in the Concise Guide to PHARMACOLOGY 2019/20.^26^, ^27^

## 3 RESULTS

### 3.1 Study population

The study was conducted at four centers in the United States between 29 October 2021 and 7 June 2022. Thirty‐six participants were enrolled and completed the study, and their demographics are shown in Table 1. The moderate hepatic impairment arm (*n* = 8) was enrolled first followed by 12 participants with normal hepatic function who were matched to the moderate hepatic impairment arm by age, sex, and BMI. After a preliminary data analysis that estimated the effect of moderate hepatic impairment on soticlestat pharmacokinetics, the mild hepatic impairment arm was enrolled. Among the 12 previously enrolled healthy participants, five also matched to the mild hepatic impairment arm, while the remaining seven participants could not be used for matching purposes to this group. As a result, seven additional healthy participants were enrolled to ensure a minimum of 12 participants with normal hepatic function and matching the mean of the mild hepatic impairment arm by age (mean ± 10 years), sex (± 2 per sex), and BMI (mean ± 10%) were enrolled. One participant was enrolled with moderate hepatic impairment at screening but was determined to have severe hepatic impairment the day before dosing. This participant was dosed and followed through the study, but their data were not included in the pharmacokinetic analyses.

**TABLE 1:** Participant demographics.

	Hepatic impairment		Controls with normal hepatic function matched to each hepatic impairment group		Normal hepatic function (*n* = 19)	Overall (*n* = 36)
	Moderate (*n* = 8)	Mild (*n* = 8)	Moderate (*n* = 12)	Mild (*n* = 12)		
Age, years						
Mean ± SD	59.4 ± 8.5	56.4 ± 10.0	60.4 ± 7.2	54.6 ± 3.9	58.2 ± 6.6	58.3 ± 7.7
Median (range)	58.5 (46–72)	57.0 (41–72)	61.5 (51–68)	53.5 (51–62)	57.0 (51–68)	57.0 (41–72)
Female, *n* (%)	3 (37.5)	2 (25.0)	7 (58.3)	5 (41.7)	11 (57.9)	16 (44.4)
Male, *n* (%)	5 (62.5)	6 (75.0)	5 (41.7)	7 (58.3)	8 (42.1)	20 (55.6)
Race, *n* (%)						
Black, native Hawaiian, or other Pacific Islander	1 (12.5)	1 (12.5)	2 (16.7)	4 (33.3)	5 (26.3)	7 (19.4)
White	7 (87.5)	7 (87.5)	10 (83.3)	8 (66.7)	14 (73.7)	29 (80.6)
Ethnicity, *n* (%)						
Hispanic or Latino	6 (75.0)	5 (62.5)	7 (58.3)	6 (50.0)	11 (57.9)	23 (63.9)
Not Hispanic or Latino	2 (25.0)	3 (37.5)	5 (41.7)	6 (50.0)	8 (42.1)	13 (36.1)
BMI, kg/m^2^						
Mean ± SD	30.3 ± 4.0	30.6 ± 4.9	29.4 ± 2.0	30.4 ± 1.3	29.8 ± 1.8	30.0 ± 3.2
Median (range)	29.7 (23.8–36.5)	30.2 (24.0–37.1)	29.1 (26.9–32.3)	30.5 (28.1–32.3)	30.1 (26.9–32.3)	29.8 (23.8–37.1)
Child‐Pugh score						
Mean ± SD	8.0 ± 0.9	5.5 ± 0.5	–	–	–	6.9 ± 1.6^a^
Median (range)	8.0 (7.0–9.0)	5.5 (5.0–6.0)	–	–	–	7.0 (5.0–10.0)

Abbreviations: BMI, body mass index; SD, standard deviation.

^a^ Total = 17.

A preliminary pharmacokinetic data analysis demonstrated an approximately 3‐fold increase in soticlestat total plasma exposure in participants with moderate hepatic impairment compared with healthy matched participants with normal hepatic function. Based on this information, a decision was taken not to recommend soticlestat dosing in patients with severe hepatic impairment. The protocol was amended and participants with severe hepatic impairment were not enrolled in the current study; instead, the impact of soticlestat was assessed in participants with mild hepatic impairment.

### 3.2 Pharmacokinetic parameters: soticlestat

**FIGURE 1:**
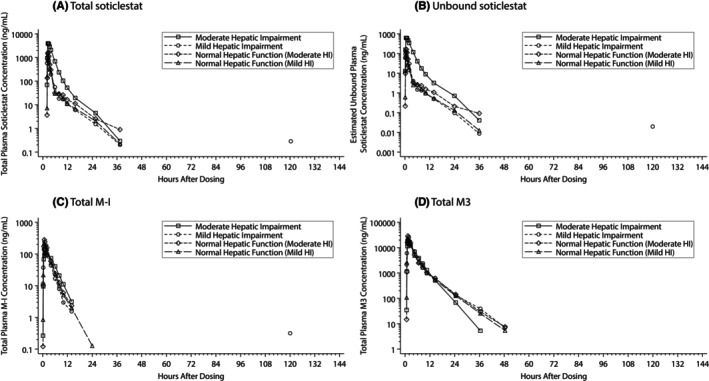
Mean plasma concentrations (semi‐log scale) of (A) total soticlestat, (B) unbound soticlestat, (C) total M‐I, and (D) total M3. HI, hepatic impairment.

Participants with moderate hepatic impairment had lower proportions of bound soticlestat and higher proportions of unbound soticlestat than participants with mild hepatic impairment and age‐matched participants with normal hepatic function. Bound and unbound soticlestat values were similar between participants with mild hepatic function and their matched participants with normal hepatic function (Table S1). Both total and unbound plasma soticlestat concentrations showed a multiphasic disposition (Figure 1; Figure S1). Total plasma soticlestat was quantifiable by 0.133 h post‐dose in most participants with moderate or mild hepatic impairment, and by 0.25 and 0.133 h post‐dose in most participants with normal hepatic function matched to the moderate and mild hepatic impairment groups, respectively.

Pharmacokinetic parameters for total and unbound plasma soticlestat are shown in Table 2, and comparisons between the hepatic impairment groups and matched groups with normal hepatic function are in Table 3. Comparison of total plasma soticlestat in participants with moderate hepatic impairment versus matched participants with normal hepatic function resulted in geometric least squares mean ratios (GMRs) of 214.93% (2.15‐fold) for *C*_max_, 316.27% (3.16‐fold) for AUC_last_, and 299.21% (2.99‐fold) for AUC_∞_. The corresponding GMRs for unbound plasma soticlestat were 420.59% (4.21‐fold), 618.89% (6.19‐fold), and 598.26% (5.98‐fold), respectively. Comparison of total plasma soticlestat in participants with mild hepatic impairment versus matched participants with normal hepatic function resulted in GMRs of 145.42% (1.45‐fold) for *C*_max_, 135.26% (1.35‐fold) for AUC_last_, and 130.25% (1.30‐fold) for AUC_∞_. The corresponding GMRs for unbound plasma soticlestat were 122.10% (1.22‐fold), 113.57% (1.14‐fold), and 113.24% (1.13‐fold), respectively.

**TABLE 2:** Pharmacokinetic parameters for total and unbound plasma soticlestat.

	Hepatic impairment		Controls with normal hepatic function matched to each hepatic impairment group	
	Moderate (*n* = 8)	Mild (*n* = 8)	Moderate (*n* = 12)	Mild (*n* = 12)
Total				
Geometric mean (geometric percent coefficient of variation) *C*_max_, ng/mL	3666 (130.9)	1435 (109.9)	1705 (81.9)	986.9 (58.0)
Geometric mean (geometric percent coefficient of variation) AUC_∞_, ng·h/mL	5760 (263.0)	1539 (67.1); *n* = 6	1925 (53.3); *n* = 10	1182 (46.3); *n* = 11
Geometric mean (geometric percent coefficient of variation) AUC_last_, ng·h/mL	5732 (266.1)	1522 (55.5)	1812 (58.1)	1126 (46.2)
Median (range) *t*_max_, h	0.500 (0.50–1.00)	0.500 (0.25–2.00)	0.625 (0.50–2.00)	0.625 (0.50–2.00)
Mean ± SD *t*_½z_, h	3.657 ± 0.9753	4.850 ± 1.9335; *n* = 6	5.007 ± 2.8323; *n* = 10	4.518 ± 2.4269; *n* = 11
Mean ± SD CL/F, L/h	178.1 ± 369.31	225.3 ± 128.83; *n* = 6	177.4 ± 112.40; *n* = 10	276.4 ± 115.90; *n* = 11
Mean ± SD V_z_/F, L	890.1 ± 1895.4	1557 ± 1002.7; *n* = 6	1110 ± 602.56; *n* = 10	1532 ± 525.98; *n* = 11
Unbound				
Geometric mean (geometric percent coefficient of variation) *C*_max_, ng/mL	621.6 (130.4)	105.0 (127.8)	147.8 (90.9)	86.00 (63.0)
Geometric mean (geometric percent coefficient of variation) AUC_∞_, ng·h/mL	976.7 (216.5)	122.2 (81.7); *n* = 6	163.3 (53.5); *n* = 10	107.9 (30.7); *n* = 11
Geometric mean (geometric percent coefficient of variation) AUC_last_, ng·h/mL	972.0 (218.8)	111.4 (71.4)	157.1 (80.1)	98.08 (42.4)
Mean ± SD CL_u_/F, L/h	777.5 ± 1348.6	2903 ± 1468.2; *n* = 6	2036 ± 896.02; *n* = 10	2902 ± 953.12; *n* = 11
Mean ± SD V_z,u_/F, L	3895 ± 6885.8	20 980 ± 16 630; *n* = 6	14 570 ± 11 187; *n* = 10	18 670 ± 10 781; *n* = 11

Abbreviations: AUC_∞_, area under the total plasma concentration‐time curve from time 0 to infinity; AUC_last_, area under the total plasma concentration‐time curve from time 0 to time of the last quantifiable total plasma concentration; CL/F, apparent clearance after extravascular administration, calculated using the observed value of the last quantifiable total plasma concentration; *C*_max_, maximum observed total plasma concentration; SD, standard deviation; *t*_½z_, terminal disposition phase half‐life; *t*_max_, time of first occurrence of maximum observed total plasma concentration; V_Z_/F, apparent volume of distribution during the terminal disposition phase after extravascular administration, calculated using the observed value of the last quantifiable total plasma concentration.

**TABLE 3:** Comparison of pharmacokinetic parameters for total and unbound plasma soticlestat in participants with moderate and mild hepatic impairment versus matched controls with normal hepatic function.

	Moderate (test)		Matched control (reference)				Cohort inter‐participant CV%	
	Geometric LSM	*n*	Geometric LSM	*n*	GMR, %	90% CI	Moderate	Matched control
Total								
*C*_max_, ng/mL	3666	8	1705	12	214.93	103.47–446.45	130.9	81.9
AUC_last_, ng·h/mL	5732	8	1812	12	316.27	117.68–849.97	266.1	58.1
AUC_∞_, ng·h/mL	5760	8	1925	10	299.21	111.75–801.17	263.0	53.3
Unbound								
*C*_max_, ng/mL	621.6	8	147.8	12	420.59	217.15–814.63	103.4	90.9
AUC_last_, ng·h/mL	972.0	8	157.1	12	618.89	244.48–1566.68	218.8	80.1
AUC_∞_, ng·h/mL	976.7	8	163.3	10	598.26	241.50–1482.02	216.5	53.5

	Mild (test)		Matched control (reference)				Cohort inter‐participant CV%	
	Geometric LSM	*n*	Geometric LSM	*n*	GMR, %	90% CI	Mild	Matched control
Total								
*C*_max_, ng/mL	1435	8	986.9	12	145.42	77.18–273.98	109.9	58.0
AUC_last_, ng·h/mL	1522	8	1126	12	135.26	91.22–200.57	55.5	46.2
AUC_∞_, ng·h/mL	1539	6	1182	11	130.25	77.02–220.26	67.1	46.3
Unbound								
*C*_max_, ng/mL	105.0	8	86.00	12	122.10	60.77–245.32	127.8	63.0
AUC_last_, ng·h/mL	111.4	8	98.08	12	113.57	71.72–179.84	71.4	42.4
AUC_∞_, ng·h/mL	122.2	6	107.9	11	113.24	62.52–205.14	81.7	30.7

Abbreviations: AUC_∞_, area under the total plasma concentration‐time curve from time 0 to infinity; AUC_last_, area under the total plasma concentration‐time curve from time 0 to time of the last quantifiable total plasma concentration; CI, confidence interval; *C*_max_, maximum observed total plasma concentration; CV%, arithmetic percent coefficient of variation; GMR, geometric least squares mean ratio; LSM, least squares mean.

### 3.3 Pharmacokinetic parameters: metabolites (M‐I and M3)

Total plasma M‐I concentrations showed mono‐ or biphasic disposition, whereas total plasma M3 concentrations showed multiphasic disposition (Figure 1; Figure S1).

Comparison of total plasma M‐I in participants with moderate hepatic impairment versus matched participants with normal hepatic function resulted in GMRs of 36.46% for *C*_max_, 73.44% for AUC_last_, and 73.87% for AUC_∞_. The corresponding GMRs for mild hepatic impairment versus matched participants with normal hepatic function were 56.93%, 74.08%, and 71.57%, respectively (Table 4; Table S2). GMRs of MPRs for *C*_max_, AUC_last_, and AUC_∞_ were 16.96%, 23.22%, and 23.22%, respectively, for moderate hepatic function, and 39.15%, 54.77%, and 54.22%, respectively, for mild hepatic function versus normal hepatic function.

**TABLE 4:** Comparison of pharmacokinetic parameters for total plasma M‐I in participants with moderate and mild hepatic impairment versus matched controls with normal hepatic function.

	Moderate (test)		Matched control (reference)				Cohort inter‐participant CV%	
	Geometric LSM	*n*	Geometric LSM	*n*	GMR, %	90% CI	Moderate	Matched control
*C*_max_, ng/mL	116.0	8	318.1	12	36.46	29.06–45.75	26.5	32.9
MPR *C*_max_	0.03034	8	0.1789	12	16.96	9.27–31.03	102.1	56.6
AUC_last_, ng·h/mL	481.1	8	655.1	12	73.44	47.63–113.23	68.8	31.1
MPR AUC_last_	0.08048	8	0.3466	12	23.22	12.42–43.40	113.9	37.0
AUC_∞_, ng·h/mL	491.7	8	665.6	12	73.87	48.00–113.68	68.5	30.5
MPR AUC_∞_	0.08185	8	0.3526	10	23.22	12.42–43.41	112.9	37.4

	Mild (test)		Matched control (reference)				Cohort inter‐participant CV%	
	Geometric LSM	*n*	Geometric LSM	*n*	GMR, %	90% CI	Mild	Matched control
*C*_max_, ng/mL	166.6	8	292.6	12	56.93	37.14–87.27	65.1	40.0
MPR *C*_max_	0.1113	8	0.2843	12	39.15	24.04–63.75	79.0	38.0
AUC_last_, ng·h/mL	436.8	8	589.6	12	74.08	55.37–99.11	37.3	38.2
MPR AUC_last_	0.2751	8	0.5023	12	54.77	39.17–76.57	51.0	24.5
AUC_∞_, ng·h/mL	428.6	7	598.9	12	71.57	52.34–97.86	38.7	37.7
MPR AUC_∞_	0.2757	6	0.5085	11	54.22	33.99–86.51	60.8	25.5

Abbreviations: AUC_∞_, area under the total plasma concentration‐time curve from time 0 to infinity; AUC_last_, area under the total plasma concentration‐time curve from time 0 to time of the last quantifiable total plasma concentration; CI, confidence interval; *C*_max_, maximum observed total plasma concentration; CV%, arithmetic percent coefficient of variation; GMR, geometric least squares mean ratio; LSM, least squares mean; MPR, metabolite‐to‐parent ratio.

Comparison of total plasma M3 in participants with moderate hepatic impairment versus matched participants with normal hepatic function resulted in GMRs of 49.89% for *C*_max_, 86.80% for AUC_last_, and 86.88%, for AUC_∞_. The corresponding GMRs for mild hepatic impairment versus matched participants with normal hepatic function were 104.26%, 105.31%, and 105.34%, respectively (Table 5; Table S3). GMRs of MPRs for *C*_max_, AUC_last_, and AUC_∞_ were 23.21%, 27.45%, and 28.57%, respectively, for moderate hepatic function and 71.70%, 77.86%, and 84.96%, respectively, for mild hepatic function versus normal hepatic function.

**TABLE 5:** Comparison of pharmacokinetic parameters for total plasma M3 in participants with moderate and mild hepatic impairment versus matched controls with normal hepatic function.

	Moderate (test)		Matched control (reference)				Cohort inter‐participant CV%	
	Geometric LSM	*n*	Geometric LSM	*n*	GMR, %	90% CI	Moderate	Matched control
*C*_max_, ng/mL	15 790	8	31 650	12	49.89	35.41–70.30	52.8	22.8
MPR *C*_max_	2.927	8	12.61	12	23.21	9.18–58.69	227.8	59.5
AUC_last_, ng·h/mL	63 280	8	72 900	12	86.80	72.63–103.75	25.3	15.9
MPR AUC_last_	7.502	8	27.33	12	27.45	9.93–75.88	290.1	48.6
AUC_∞_, ng·h/mL	63 470	8	73 060	12	86.88	72.73–103.78	25.2	15.8
MPR AUC_∞_	7.489	8	26.21	10	28.57	10.36–78.83	286.6	47.8

	Mild (test)		Matched control (reference)				Cohort inter‐participant CV%	
	Geometric LSM	*n*	Geometric LSM	*n*	GMR, %	90% CI	Mild	Matched control
*C*_max_, ng/mL	28 960	8	27 770	12	104.26	83.44–130.28	27.9	29.4
MPR *C*_max_	13.71	8	19.12	12	71.70	43.56–118.01	79.9	43.2
AUC_last_, ng·h/mL	71 220	8	67 630	12	105.31	82.45–134.52	35.8	19.6
MPR AUC_last_	31.79	8	40.83	12	77.86	49.14–123.35	72.6	38.8
AUC_∞_, ng·h/mL	71 430	8	67 810	12	105.34	82.47–134.55	35.8	19.5
MPR AUC_∞_	33.72	6	39.69	11	84.96	45.51–158.59	86.2	39.8

Abbreviations: AUC_∞_, area under the total plasma concentration‐time curve from time 0 to infinity; AUC_last_, area under the total plasma concentration‐time curve from time 0 to time of the last quantifiable total plasma concentration; CI, confidence interval; *C*_max_, maximum observed total plasma concentration; CV%, arithmetic percent coefficient of variation; GMR, geometric least squares mean ratio; LSM, least squares mean; MPR, metabolite‐to‐parent ratio.

### 3.4 Safety

Five participants experienced a total of six TEAEs, as shown in Table 6. All TEAEs were mild and had resolved by the end of the study, and two were considered to be study drug related. There were no TEAEs based on serum chemistry, hematology, coagulation, urinalysis, vital signs, ECG, or Columbia‐Suicide Severity Rating Scale.

**TABLE 6:** Treatment‐emergent adverse events.

	Hepatic impairment			Matched controls with normal hepatic function (*n* = 19)	Overall (*n* = 36)
	Severe (*n* = 1)	Moderate (*n* = 8)	Mild (*n* = 8)		
Participants with a TEAE, *n* (%)	1 (100)	1 (12.5)	1 (12.5)	2 (10.5)	5 (13.9)
Constipation	0	0	0	1 (5.3)	1 (2.8)
Alpha‐1 acid glycoprotein increased	0	0	0	1 (5.3)	1 (2.8)
Dizziness	0	1 (12.5)	0	0	1 (2.8)
Headache	0	0	1 (12.5)	0	1 (2.8)
Cough	1 (100)	0	0	0	1 (2.8)
Epistaxis	0	1 (12.5)	0	0	1 (2.8)

Abbreviation: TEAE, treatment‐emergent adverse event.

## 4 DISCUSSION

The liver is involved in the clearance of many medications, and hepatic impairment can reduce the liver's metabolic capacity through altered physiological parameters such as a decreased abundance of drug‐metabolizing enzymes, decreased functional hepatic volume, decreased hepatic blood flow, and decreased drug‐binding plasma proteins. For drugs primarily metabolized by the liver, hepatic impairment can lead to decreased clearance and increased exposure. The degree of impact on drug metabolism depends on the severity of liver dysfunction, with more severe impairment generally causing greater reductions in metabolic capacity.^28^, ^29^, ^30^, ^31^ Metabolism is the main elimination pathway for soticlestat,^22^ and therefore this study was conducted to evaluate the impact of hepatic impairment on soticlestat pharmacokinetics to inform dosing decisions in patients with hepatic impairment. A preliminary physiology‐based pharmacokinetic model and simulation predicted a <50% decrease in CL/F in all the hepatic impairment groups (mild, moderate, and severe) versus the group with normal hepatic function. Based on the model predictions, the pharmacokinetics of total plasma soticlestat in participants with moderate hepatic impairment and healthy matched participants with normal hepatic function were assessed first, and a preliminary analysis was conducted.^24^ However, the preliminary model predictions underestimated the impact of moderate hepatic impairment on soticlestat plasma exposure, likely because some of the model input parameters, such as the faction of soticlestat metabolized by each metabolic enzyme, were not available at the time of the analysis. The preliminary analysis indicated a higher impact of moderate hepatic impairment on total plasma soticlestat than predicted, with AUC_∞_ and AUC_last_ values approximately 3‐fold higher compared with healthy participants with normal hepatic function. As a result, a decision was made not to recommend soticlestat administration in patients with severe hepatic impairment, and participants with severe hepatic impairment were not included in the study. Instead, the impact of mild hepatic impairment on the pharmacokinetics of soticlestat was investigated.

The pharmacokinetic parameters of soticlestat in individuals with normal hepatic function were similar to those reported in other soticlestat phase 1 studies of healthy persons,^16^, ^17^, ^32^ although the participants are older in this study, especially those matched to participants with moderate hepatic impairment. Compared with healthy individuals matched to participants with mild hepatic impairment, higher age (median, 61.5 vs. 53.5 years) may have contributed to higher exposures observed in these individuals (geometric mean AUC_∞_, 1925 vs. 1182 ng●h/mL).

Total plasma soticlestat PK parameters (primary endpoints; *C*_max_, AUC_last_, and AUC_∞_) were approximately 115%, 216%, and 199% higher with moderate hepatic impairment and approximately 45%, 35%, and 30% higher with mild hepatic impairment, respectively, than healthy matched participants with normal hepatic function. As introduced earlier, soticlestat was mainly bound to human α‐1‐acid glycoprotein. Decreased α‐1‐acid glycoprotein levels have been observed in patients with liver cirrhosis compared with healthy controls. The severity of hepatic impairment was negatively correlated with α‐1‐acid glycoprotein concentration.^33^, ^34^, ^35^, ^36^ As expected, moderate hepatic impairment caused decreased binding of soticlestat to plasma proteins compared with healthy matched participants (unbound fraction: 17.5% vs. 9.68%, respectively) and had an even greater impact on unbound plasma soticlestat exposure. Mild hepatic impairment had a negligible impact on protein binding (unbound fraction: 7.99% vs. 9.36%, respectively) resulting in similar unbound plasma soticlestat exposure to that of participants with normal hepatic function.

As discussed earlier, for drugs such as soticlestat that are primarily metabolized by the liver, hepatic impairment can lead to decreased clearance and increased exposure. As expected, moderate hepatic impairment appears to decrease the liver's ability to metabolize soticlestat to M‐I, and mild hepatic impairment also resulted in lower total plasma M‐I exposure. M‐I C_max_, AUC_last_, and AUC_∞_ were approximately 64%, 27%, and 26% lower with moderate hepatic impairment and approximately 43%, 26%, and 28% lower with mild hepatic impairment, respectively, than healthy matched participants with normal hepatic function. This was also reflected by the MPR *C*_max_, MPR AUC_last_, and MPR AUC_∞_ values that were approximately 83%, 77%, and 77% lower with moderate hepatic impairment and approximately 61%, 45%, and 46% lower with mild hepatic impairment, respectively, compared with healthy matched participants with normal hepatic function.

Glucuronidation of soticlestat was affected to some extent by moderate but not by mild hepatic impairment. M3 *C*_max_, AUC_last_, and AUC_∞_ were approximately 50%, 13%, and 13% lower with moderate hepatic impairment and approximately 4%, 5%, and 5% higher with mild hepatic impairment, respectively, than healthy matched participants with normal hepatic function. This was also reflected by the MPR *C*_max_, MPR AUC_last_, and MPR AUC_∞_ values that were approximately 77%, 73%, and 71% lower with moderate hepatic impairment and approximately 28%, 22%, and 15% lower with mild hepatic impairment, respectively, compared with healthy matched participants with normal hepatic function. Soticlestat is known to be predominantly metabolized by the liver via the glucuronidation pathway, with <1% excreted in urine.^16^ M3 is the major metabolite mediated by uridine 5′‐diphospho‐glucuronosyltransferase (UGT)1A9 and UGT2B4, with these enzymes accounting for approximately 90% of soticlestat metabolism. The N‐oxide metabolite (M‐I) is formed by CYP3A4.^18^, ^22^ Considering the metabolic pathway, as discussed earlier, it was expected that moderate hepatic impairment would decrease the liver's ability to metabolize soticlestat to metabolites. Mild hepatic impairment only had an impact on M‐I and not on glucuronidation of soticlestat via UGT2B4 and UGT1A9; the lack of impact on glucuronidation may be due to the large UGT capacity in the liver.

The metabolites (M‐I and M3) were measured in this study to gain an estimate of the effect of hepatic impairment on the liver's ability to metabolize soticlestat to these metabolites. However, any impact on the exposure of these metabolites is not clinically relevant; the M‐I metabolite displayed only weak CH24H inhibition that was 203‐fold lower than for soticlestat (IC_50_: 913.5 nmol/L for M‐I; 4.5 nmol/L for soticlestat) while the M3 metabolite is inactive. As such, the assessment of dose adjustment will be based solely on the plasma exposure to the parent drug, soticlestat.

TEAEs were similar across the study arms, all were mild, and no new safety findings were observed in the study.

This was a multicenter study with no statistical adjustments for multiple comparisons, which could be considered a limitation. In addition, the sample size is small for each of the groups. However, the chosen sample size was considered adequate to characterize pharmacokinetics in the different groups and is consistent with regulatory guidelines.^24^

This phase 1, open‐label study was conducted to compare the pharmacokinetics and safety of a single oral dose of soticlestat 300 mg in participants with moderate or mild hepatic impairment versus matched individuals with normal hepatic function. All observed TEAEs were mild and no new safety findings were observed. However, based on the pharmacokinetic results, a dose reduction will be required (approximately 6‐fold decrease based on increase in unbound plasma soticlestat AUCs) for individuals with moderate hepatic impairment, and soticlestat administration is not recommended in patients with severe hepatic impairment. No dose reduction of soticlestat is required for persons with mild hepatic impairment as any increase in unbound plasma soticlestat AUCs is negligible.

## AUTHOR CONTRIBUTIONS

All authors contributed to the study's conception and design. Data analysis was performed by Clinical Pharmacology and Biostatistics. All authors were involved in manuscript preparation and review. All authors have read and agreed to the final version of the manuscript.

## FUNDING INFORMATION

This research was funded by Takeda Development Center Americas, Inc., who also provided open‐access funding.

## CONFLICT OF INTEREST STATEMENT

Wei Yin, Pranab Mitra, Veronique Copalu, Valerie Lloyd, Mike Baratta, Mahnaz Asgharnejad, Tom Hui, and Yasir Khan are employees of Takeda Development Center Americas, Inc., and hold stock in Takeda Pharmaceuticals Company Limited. Thomas C. Marbury is an employee and equity owner of Orlando Clinical Research Center. Juan Carlos Rondon is an employee of Clinical Pharmacology of Miami, LLC. Eric J. Lawitz reports being a researcher for 89Bio, AbbVie, Akero Therapeutics, Alnylam, Amgen, AstraZeneca, Axcella Health, Boehringer Ingelheim, Bristol Myers Squibb, Cymabay Therapeutics, DSM, Eli Lilly, Enanta, Enyo Pharma, Exalenz Bioscience, Galectin Therapeutics, Galmed, Genentech, Genfit, Gilead, GlaxoSmithKline, Hanmi, Hightide Biopharma, Intercept, Inventiva, Janssen, Madrigal, Merck, NGM Biopharmaceuticals, Northsea Therapeutics, Novartis, Novo Nordisk, Pfizer, Poxel, Roche, Sagimet Biosciences, Takeda, Terns, Viking Therapeutics, and Zydus; an advisor for Akero, Boehringer Ingelheim, Bristol Myers Squibb, Intercept, Novo Nordisk, Sagimet, and Terns; and a speaker for AbbVie, Gilead, and Intercept.

## ETHICS STATEMENT

All study documents were reviewed by the Advarra Institutional Review Board (Columbia, MD, USA) prior to study initiation. The study was approved by the local Institutional Review Board of the study sites and conducted in accordance with the Declaration of Helsinki, International Conference on Harmonization Harmonized Tripartite Guideline for Good Clinical Practice, and all applicable regulations. All participants provided written informed consent.

## CLINICAL TRIAL REGISTRATION

EudraCT, 2021–006373‐29; ClinicalTrials.gov, NCT05098054.

## PRINCIPAL INVESTIGATOR STATEMENT

The authors confirm that the Principal Investigators for this paper are Thomas C. Marbury, Juan Carlos Rondon, and Eric J. Lawitz and that they had direct clinical responsibility for participants.

## Supporting information


**Data S1:** Supporting Information.

## Data Availability

The datasets, including the redacted study protocol, redacted statistical analysis plan, and individual participants' data supporting the results of the study, will be made available after the publication of study results within 3 months from initial request to researchers who provide a methodologically sound proposal. The data will be provided after its de‐identification, in compliance with applicable privacy laws, data protection, and requirements for consent and anonymization.

## References

[1] Wirrell EC , Nabbout R , Scheffer IE , et al. Methodology for classification and definition of epilepsy syndromes with list of syndromes: report of the ILAE task force on nosology and definitions. Epilepsia. 2022;63(6):1333‐1348. doi:10.1111/epi.17237 35503715

[2] Andrade DM , Nascimento FA . Dravet syndrome: Genetics, clinical features, and diagnosis. Accessed 13 June 2023. https://www.uptodate.com/contents/dravet‐syndrome‐genetics‐clinical‐features‐and‐diagnosis?search=Dravet&source=search_result&selectedTitle=2~28&usage_type=default&display_rank=2

[3] Wilfong A . Lennox‐Gastaut syndrome. Accessed 13 June 2023. https://www.uptodate.com/contents/lennox‐gastaut‐syndrome?search=lennox%20gastaut%20syndrome&source=search_result&selectedTitle=1~150&usage_type=default&display_rank=1

[4] Wu J , Zhang L , Zhou X , et al. Efficacy and safety of adjunctive antiseizure medications for Dravet syndrome: a systematic review and network meta‐analysis. Front Pharmacol. 2022;13:980937. doi:10.3389/fphar.2022.980937 36120377 PMC9471196

[5] Strzelczyk A , Schubert‐Bast S . Expanding the treatment landscape for Lennox‐Gastaut syndrome: current and future strategies. CNS Drugs. 2021;35(1):61‐83. doi:10.1007/s40263-020-00784-8 33479851 PMC7873005

[6] Nishi T , Metcalf CS , Fujimoto S , et al. Anticonvulsive properties of soticlestat, a novel cholesterol 24‐hydroxylase inhibitor. Epilepsia. 2022;63(6):1580‐1590. doi:10.1111/epi.17232 35316533 PMC9311151

[7] A Study of Soticlestat as an Add‐on Therapy in Children and Young Adults With Dravet Syndrome. Accessed 12 June 2023. Clinicaltrials.gov, https://clinicaltrials.gov/ct2/show/NCT04940624

[8] A Study of Soticlestat as an Add‐on Therapy in Children, Teenagers, and Adults With Lennox‐Gastaut Syndrome. Accessed 12 June 2023. Clinicaltrials.gov, https://clinicaltrials.gov/ct2/show/NCT04938427

[9] Paul SM , Doherty JJ , Robichaud AJ , et al. The major brain cholesterol metabolite 24(S)‐hydroxycholesterol is a potent allosteric modulator of N‐methyl‐D‐aspartate receptors. J Neurosci. 2013;33(44):17290‐17300. doi:10.1523/JNEUROSCI.2619-13.2013 24174662 PMC3812502

[10] Gc JB , Chen J , Pokharel SM , et al. Molecular basis for the recognition of 24‐(S)‐hydroxycholesterol by integrin alphavbeta3. Sci Rep. 2023;13(1):9166. doi:10.1038/s41598-023-36040-4 37280310 PMC10244445

[11] Nishi T , Kondo S , Miyamoto M , et al. Soticlestat, a novel cholesterol 24‐hydroxylase inhibitor shows a therapeutic potential for neural hyperexcitation in mice. Sci Rep. 2020;10(1):17081. doi:10.1038/s41598-020-74036-6 33051477 PMC7553946

[12] Barker‐Haliski M , Nishi T , White HS . Soticlestat, a novel cholesterol 24‐hydroxylase inhibitor, modifies acute seizure burden and chronic epilepsy‐related behavioral deficits following Theiler's virus infection in mice. Neuropharmacology. 2023;222:109310. doi:10.1016/j.neuropharm.2022.109310 36341806

[13] Hawkins NA , Jurado M , Thaxton TT , et al. Soticlestat, a novel cholesterol 24‐hydroxylase inhibitor, reduces seizures and premature death in Dravet syndrome mice. Epilepsia. 2021;62(11):2845‐2857. doi:10.1111/epi.17062 34510432 PMC9291096

[14] Salamone A , Terrone G , Di Sapia R , et al. Cholesterol 24‐hydroxylase is a novel pharmacological target for anti‐ictogenic and disease modification effects in epilepsy. Neurobiol Dis. 2022;173:105835. doi:10.1016/j.nbd.2022.105835 35932989

[15] Koike T , Yoshikawa M , Ando HK , et al. Discovery of Soticlestat, a potent and selective inhibitor for cholesterol 24‐hydroxylase (CH24H). J Med Chem. 2021;64(16):12228‐12244. doi:10.1021/acs.jmedchem.1c00864 34387987

[16] Wang S , Chen G , Merlo Pich E , Affinito J , Cwik M , Faessel H . Safety, tolerability, pharmacokinetics, pharmacodynamics, bioavailability and food effect of single doses of soticlestat in healthy subjects. Br J Clin Pharmacol. 2021;87(11):4354‐4365. doi:10.1111/bcp.14854 33837574 PMC8597018

[17] Wang S , Chen G , Merlo Pich E , Affinito J , Cwik M , Faessel HM . Pharmacokinetics, pharmacodynamics and safety assessment of multiple doses of soticlestat in healthy volunteers. Br J Clin Pharmacol. 2022;88(6):2899‐2908. doi:10.1111/bcp.15225 35001412 PMC9305210

[18] Yin W , Facius A , Wagner T , et al. Population pharmacokinetics, enzyme occupancy, and 24S‐hydroxycholesterol modeling of soticlestat, a novel cholesterol 24‐hydroxylase inhibitor, in healthy adults. Clin Transl Sci. 2023;16(7):1149‐1162. doi:10.1111/cts.13517 37212649 PMC10339692

[19] Halford JJ , Sperling MR , Arkilo D , et al. A phase 1b/2a study of soticlestat as adjunctive therapy in participants with developmental and/or epileptic encephalopathies. Epilepsy Res. 2021;174:106646. doi:10.1016/j.eplepsyres.2021.106646 33940389

[20] Demarest S , Jeste S , Agarwal N , et al. Efficacy, safety, and tolerability of soticlestat as adjunctive therapy for the treatment of seizures in patients with Dup15q syndrome or CDKL5 deficiency disorder in an open‐label signal‐finding phase II study (ARCADE). Epilepsy Behav. 2023;142:109173. doi:10.1016/j.yebeh.2023.109173 37011526

[21] Hahn CD , Jiang Y , Villanueva V , et al. A phase 2, randomized, double‐blind, placebo‐controlled study to evaluate the efficacy and safety of soticlestat as adjunctive therapy in pediatric patients with Dravet syndrome or Lennox‐Gastaut syndrome (ELEKTRA). Epilepsia. 2022;63(10):2671‐2683. doi:10.1111/epi.17367 35841234 PMC9804149

[22] Yin W , Ballard TE , Zhu X , et al. Investigation of the absolute bioavailability, mass balance, metabolism, and excretion of the cholesterol 24‐hydroxylase inhibitor soticlestat in healthy volunteers. Br J Clin Pharmacol. 2024;90(2):516‐527. doi:10.1111/bcp.15917 37771051

[23] Rodighiero V . Effects of liver disease on pharmacokinetics. An update. Clin Pharmacokinet. 1999;37(5):399‐431. doi:10.2165/00003088-199937050-00004 10589374

[24] US Food and Drug Administration . Guidance for industry pharmacokinetics in patients with impaired hepatic function: Study design, data analysis, and impact on dosing and labeling. Accessed 12 June 2023. https://www.fda.gov/media/71311/download

[25] A Study of Soticlestat in Adults With Liver Failure Compared to Those With Normal Liver Function. Accessed 12 June 2023. Clinicaltrials.gov, https://clinicaltrials.gov/ct2/show/NCT05098054

[26] Harding SD , Sharman JL , Faccenda E , et al. The IUPHAR/BPS guide to pharmacology in 2018: updates and expansion to encompass the new guide to immunopharmacology. Nucleic Acids Res. 2018;46(D1):D1091‐D1106. doi:10.1093/nar/gkx1121 29149325 PMC5753190

[27] Alexander SPH , Fabbro D , Kelly E , et al. The concise guide to PHARMACOLOGY 2023/24: enzymes. Br J Pharmacol. 2023;180(S2):S289‐S373. doi:10.1111/bph.16181 38123154

[28] Ladumor MK , Storelli F , Liang X , et al. Predicting changes in the pharmacokinetics of CYP3A‐metabolized drugs in hepatic impairment and insights into factors driving these changes. CPT Pharmacometrics Syst Pharmacol. 2023;12(2):261‐273. doi:10.1002/psp4.12901 36540952 PMC9931433

[29] Chattopadhyay N , Riecke K , Ligges S , Zimmermann T , Halabi A , Schultze‐Mosgau MH . Effect of hepatic impairment on the pharmacokinetics of vilaprisan: an open‐label, single‐dose, parallel‐group study. Br J Clin Pharmacol. 2019;85(9):2011‐2021. doi:10.1111/bcp.13992 31112623 PMC6710501

[30] Palatini P , De Martin S . Pharmacokinetic drug interactions in liver disease: an update. World J Gastroenterol. 2016;22(3):1260‐1278. doi:10.3748/wjg.v22.i3.1260 26811663 PMC4716036

[31] Duthaler U , Bachmann F , Suenderhauf C , et al. Liver cirrhosis affects the pharmacokinetics of the six substrates of the Basel phenotyping cocktail differently. Clin Pharmacokinet. 2022;61(7):1039‐1055. doi:10.1007/s40262-022-01119-0 35570253 PMC9287224

[32] Yin W , Dong C , Stevenson A , et al. Effects of strong inhibition of cytochrome P450 3A and UDP glucuronosyltransferase 1A9 and strong induction of cytochrome P450 3A on the pharmacokinetics, safety, and tolerability of Soticlestat: two drug‐drug interaction studies in healthy volunteers. Drug Metab Dispos. 2024;52(3):180‐187. doi:10.1124/dmd.123.001444 38123352

[33] Al‐Qassabi J , Tan SPF , Phonboon P , Galetin A , Rostami‐Hodjegan A , Scotcher D . Facing the facts of altered plasma protein binding: do current models correctly predict changes in fraction unbound in special populations? J Pharm Sci. 2024;113(6):1664–1673. doi:10.1016/j.xphs.2024.02.024 38417790

[34] Roberts JA , Pea F , Lipman J . The clinical relevance of plasma protein binding changes. Clin Pharmacokinet. 2013;52(1):1‐8. doi:10.1007/s40262-012-0018-5 23150213

[35] Barre J , Houin G , Rosenbaum J , Zini R , Dhumeaux D , Tillement JP . Decreased alpha 1‐acid glycoprotein in liver cirrhosis: consequences for drug protein binding. Br J Clin Pharmacol. 1984;18(4):652‐653. doi:10.1111/j.1365-2125.1984.tb02525.x 6487513 PMC1463634

[36] Barry M , Keeling PW , Weir D , Feely J . Severity of cirrhosis and the relationship of alpha 1‐acid glycoprotein concentration to plasma protein binding of lidocaine. Clin Pharmacol Ther. 1990;47(3):366‐370. doi:10.1038/clpt.1990.41 2311337

