# Supplementary material for: Phase 1 pharmacokinetic and safety study of soticlestat in participants with mild or moderate hepatic impairment or normal hepatic function
Source: Pharmacol Res Perspect. 2024 Jul 11;12(4):e1213. doi: 10.1002/prp2.1213 (PMC11239955; doi:10.1002/prp2.1213)
Supplement: Supplementary file 1 — Data S1: Supporting Information. [file PRP2-12-e1213-s001.docx]

Supporting Information

**FIGURE S1** Mean plasma concentrations (SD; linear scale) of (A) total soticlestat, (B) unbound soticlestat, (C) total M-I, and (D) total M3. HI, hepatic impairment.


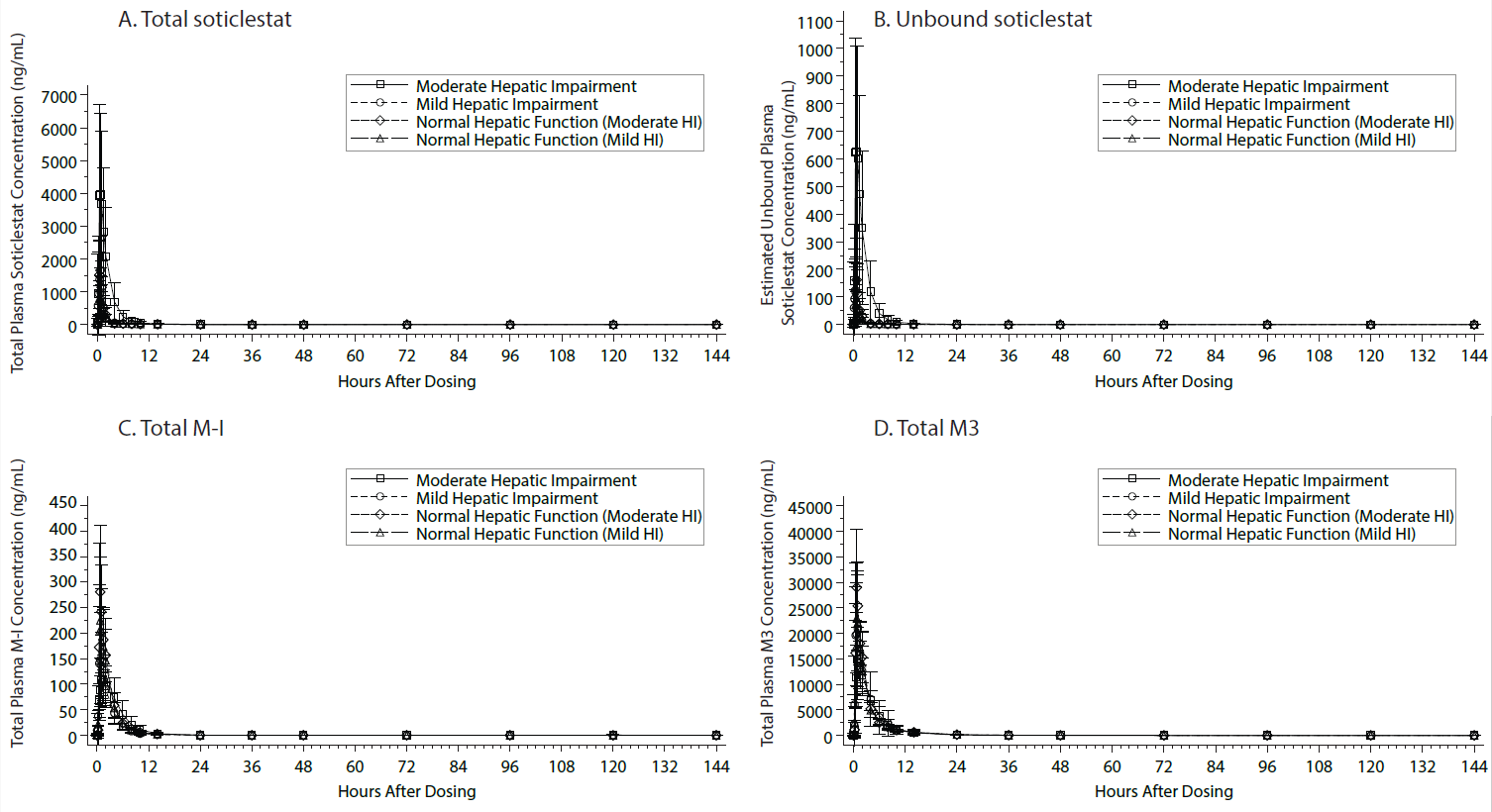
**TABLE S1** Plasma protein binding of soticlestat.

|  | **Hepatic impairment** | | | | **Controls with normal hepatic function matched to each hepatic impairment group** | | | |
| --- | --- | --- | --- | --- | --- | --- | --- | --- |
|  | **Moderate** | | **Mild** | | **Moderate** | | **Mild** | |
|  | **Bound, %  (n = 8)** | **Unbound, % (n = 8)** | **Bound, %  (n = 8)** | **Unbound, % (n = 8)** | **Bound, %  (n = 12)** | **Unbound, % (n = 12)** | **Bound, %  (n = 12)** | **Unbound, % (n = 12)** |
| Mean | 82.52 | 17.48 | 92.01 | 7.991 | 90.32 | 9.678 | 90.64 | 9.358 |
| Standard deviation | 4.7098 | 4.7098 | 3.7004 | 3.7004 | 4.6695 | 4.6695 | 3.7542 | 3.7542 |
| Minimum | 73.1 | 10.9 | 84.8 | 4.22 | 82.1 | 3.66 | 82.5 | 4.90 |
| Median | 83.02 | 16.98 | 92.70 | 7.300 | 90.59 | 9.414 | 91.63 | 8.369 |
| Maximum | 89.1 | 26.9 | 95.8 | 15.2 | 96.3 | 17.9 | 95.1 | 17.5 |
| Geometric mean | 82.39 | 16.96 | 91.94 | 7.317 | 90.21 | 8.666 | 90.57 | 8.714 |
| Geometric percent coefficient of variation | 5.9 | 26.9 | 4.1 | 46.7 | 5.3 | 53.2 | 4.2 | 40.9 |

**TABLE S2** Pharmacokinetic parameters for total plasma M-I.

|  | **Hepatic impairment** | | **Controls with normal hepatic function matched to each hepatic impairment group** | |
| --- | --- | --- | --- | --- |
|  | **Moderate (n = 8)** | **Mild (n = 8)** | **Moderate (n = 12)** | **Mild (n = 12)** |
| Median (range) t_max_, h | 1.000 (0.50-4.00) | 0.750 (0.50-4.00) | 0.750 (0.50-2.00) | 0.750 (0.50-2.00) |
| Geometric mean (geometric percent coefficient of variation) C_max_, ng/mL | 116.0 (26.5) | 166.6 (65.1) | 318.1 (32.9) | 292.6 (40.0) |
| Geometric mean (geometric percent coefficient of variation) MPR C_max_ | 0.03034 (102.1) | 0.1113 (79.0) | 0.1789 (56.6) | 0.2843 (38.0) |
| Geometric mean (geometric percent coefficient of variation) AUC_last_, ng●h/mL | 481.1 (68.8) | 436.8 (37.3) | 655.1 (31.1) | 589.6 (38.2) |
| Geometric mean (geometric percent coefficient of variation) MPR AUC_last_ | 0.08048 (113.9) | 0.2751 (51.0) | 0.3466 (37.0) | 0.5023 (24.5) |
| Geometric mean (geometric percent coefficient of variation) AUC_∞_, ng●h/mL | 491.7 (68.5) | 428.6 (38.7); n = 7 | 665.6 (30.5) | 598.9 (37.7) |
| Geometric mean (geometric percent coefficient of variation) MPR AUC_∞_ | 0.08185 (112.9) | 0.2757 (60.8); n = 6 | 0.3526 (37.4); n = 10 | 0.5085 (25.5); n = 11 |
| Mean ± SD t_½z_, h | 2.292 ± 0.3777 | 2.521 ± 1.2432; n = 7 | 2.655 ± 0.6899 | 2.844 ± 1.0283 |

Abbreviations: AUC_∞_, area under the total plasma concentration-time curve from time 0 to infinity; AUC_last_, area under the total plasma concentration-time curve from time 0 to time of the last quantifiable total plasma concentration; C_max_, maximum observed total plasma concentration; MPR, metabolite-to-parent ratio; SD, standard deviation; t_½z_, terminal disposition phase half-life; t_max_, time of first occurrence of maximum observed total plasma concentration.

**TABLE S3** Pharmacokinetic parameters for total plasma M3.

|  | **Hepatic impairment** | | **Controls with normal hepatic function matched to each hepatic impairment group** | |
| --- | --- | --- | --- | --- |
|  | **Moderate (n = 8)** | **Mild (n = 8)** | **Moderate (n = 12)** | **Mild (n = 12)** |
| Median (range) t_max_, h | 1.250 (0.50-2.00) | 0.625 (0.50-4.00) | 0.750 (0.50-2.00) | 0.750 (0.50-2.00) |
| Geometric mean (geometric percent coefficient of variation) C_max_, ng/mL | 15 790 (52.8) | 28 960 (27.9) | 31 650 (22.8) | 27 770 (29.4) |
| Geometric mean (geometric percent coefficient of variation) MPR C_max_ | 2.927 (227.8) | 13.71 (79.9) | 12.61 (59.5) | 19.12 (43.2) |
| Geometric mean (geometric percent coefficient of variation) AUC_last_, ng●h/mL | 63 280 (25.3) | 71 220 (35.8) | 72 900 (15.9) | 67 630 (19.6) |
| Geometric mean (geometric percent coefficient of variation) MPR AUC_last_ | 7.502 (290.1) | 31.79 (72.6) | 27.33 (48.6) | 40.83 (38.8) |
| Geometric mean (geometric percent coefficient of variation) AUC_∞_, ng●h/mL | 63 470 (25.2) | 71 430 (35.8) | 73 060 (15.8) | 67 810 (19.5) |
| Geometric mean (geometric percent coefficient of variation) MPR AUC_∞_ | 7.489 (286.6) | 33.72 (86.2); n = 6 | 26.21 (47.8); n = 10 | 39.69 (39.8); n = 11 |
| Mean ± SD t_½z_, h | 3.432 ± 0.6933 | 4.167 ± 1.9818 | 5.106 ± 1.4117 | 4.969 ± 1.4742 |

Abbreviations: AUC_∞_, area under the total plasma concentration-time curve from time 0 to infinity; AUC_last_, area under the total plasma concentration-time curve from time 0 to time of the last quantifiable total plasma concentration; C_max_, maximum observed total plasma concentration; MPR, metabolite-to-parent ratio; SD, standard deviation; t_½z_, terminal disposition phase half-life; t_max_, time of first occurrence of maximum observed total plasma concentration.
